# Supplementary material for: Proteotranscriptomic Profiling of the Toxic Mucus of Kulikovia alborostrata (Pilidiophora, Nemertea)
Source: Toxins (Basel). 2024 Dec 26;17(1):5. doi: 10.3390/toxins17010005 (PMC11768458; doi:10.3390/toxins17010005)
Supplement: Supplementary file 1 [file toxins-17-00005-s001.zip › Supplementary File S4.zip!/ORF 076246 LINALB/5646dc0998f06131/dom_report.pdf]

Phyre2

Email	vas9i-kz@mail.ru
Description	putative_toxin_cistein_rich_
Date	Wed Apr 5 07:02:50 BST 2023
Unique Job ID	5646dc0998f06131

Domain analysis

Rank	Aligned region
1	c6enaA_
2	d1h5oa_
3	c1z99A_
4	d1vtxa_
